# Future-Proofing Potato for Drought and Heat Tolerance by Overexpression of Hexokinase and SP6A

**DOI:** 10.3389/fpls.2020.614534

**Published:** 2021-01-12

**Authors:** Günter G. Lehretz, Sophia Sonnewald, Nitsan Lugassi, David Granot, Uwe Sonnewald

**Affiliations:** ^1^Division of Biochemistry, Department of Biology, Friedrich-Alexander-University Erlangen-Nuremberg, Erlangen, Germany; ^2^The Volcani Center, Institute of Plant Sciences, Agricultural Research Organization, Rishon Le-Zion, Israel

**Keywords:** potato, climate change, heat, drought, SP6A, Hexokinase, tuberization, combined stress

## Abstract

Crop yield is largely affected by global climate change. Especially periods of heat and drought limit crop productivity worldwide. According to current models of future climate scenarios, heatwaves and periods of drought are likely to increase. Potato, as an important food crop of temperate latitudes, is very sensitive to heat and drought which impact tuber yield and quality. To improve abiotic stress resilience of potato plants, we aimed at co-expressing hexokinase 1 from *Arabidopsis thaliana* (*AtHXK1*) in guard cells and SELF-PRUNING 6A (*SP6A*) using the leaf/stem-specific StLS1 promoter in order to increase water use efficiency as well as tuberization under drought and heat stress. Guard cell-specific expression of *AtHXK1* decreased stomatal conductance and improved water use efficiency of transgenic potato plants as has been shown for other crop plants. Additionally, co-expression with the FT-homolog *SP6A* stimulated tuberization and improved assimilate allocation to developing tubers under control as well as under single and combined drought and heat stress conditions. Thus, co-expression of both proteins provides a novel strategy to improve abiotic stress tolerance of potato plants.

## Introduction

Global climate change has become a huge threat for food security worldwide (Birch et al., 2012; Mittler et al., 2012; Fahad et al., 2017; George et al., 2017; Lamaoui et al., 2018; Dahal et al., 2019). In particular rising temperatures and reduced water availability are challenging agriculture worldwide, especially in the northern hemisphere where most cultivated plants are not adapted to such conditions (Lafta and Lorenzen, 1995; Monneveux et al., 2013; George et al., 2017). One of them is the important and widely used crop potato (*Solanum tuberosum* L.). Potato plants are very sensitive to elevated temperatures (Lafta and Lorenzen, 1995; Levy and Veilleux, 2007; Hastilestari et al., 2018; Trapero-Mozos et al., 2018), but also drought susceptible (Deblonde and Ledent, 2001). They originate from relatively cool regions in the Andes of South America and produce starchy storage organs, the tubers, which form from underground stems, the stolons. Formation of tubers naturally occurs at the end of summer under SD conditions. This process has been described previously (Hannapel et al., 2017), and is amongst various other regulators mainly controlled by a FLOWERING LOCUS T homolog (Navarro et al., 2011). In potato, this is referred to as SELF-PRUNING 6A (SP6A) and its expression correlates with tuber formation (Abelenda et al., 2011, 2014; Navarro et al., 2011, 2015; Teo et al., 2017; Lehretz et al., 2019). Besides the day length-dependent accumulation of *SP6A*, its expression is also under temperature control. Elevated temperatures result in down-regulation of *SP6A* expression, which correlates with decreased tuber yield (Hancock et al., 2014; Hastilestari et al., 2018). Recently a small RNA induced under heat and targeting *SP6A* was discovered as the underlying molecular mechanism (Lehretz et al., 2019).

Moreover, drought is an abiotic stress predicted to rise in the near future and thus harming yields (Monneveux et al., 2013). In potato, drought negatively affects plant growth, tuber number, tuber size and tuber bulking (Deblonde and Ledent, 2001; Schafleitner et al., 2007). Drought is often accompanied by heat and both together strongly decrease tuber yield (Schafleitner et al., 2007). However, even under ambient conditions lower transpiration and thus lower water consumption would be desirable to save water expenses. Previous work showed that guard cell specific overexpression of hexokinase 1 from *Arabidopsis thaliana* (*AtHXK1*) using the KST1 promoter from potato efficiently reduces transpiration and increases water use efficiency (WUE) in several crop plants including tomato and citrus (Kelly et al., 2013, 2017; Lugassi et al., 2015).

However, up to now only few reports show an improved tuber yield under heat or drought stress in potato. For example repression of TOC1 or overexpression of Hsc70 increased heat tolerance (Trapero-Mozos et al., 2018; Morris et al., 2019), whereas overexpression of a MYB or a bZIP transcription factor ameliorated drought tolerance (Shin et al., 2011; Moon et al., 2015). Improved yield under combined drought and heat stress has not been reported so far. Here, we aimed to enhance tuberization and to reduce water loss concurrently by creating transgenic potato plants overexpressing both *SP6A* and *AtHXK1.* Thereby, we achieved significant yield improvements under single as well as combined stress conditions. These transgenic plants exhibited reduced transpiration and enhanced tuberization under control conditions, but most importantly, yield reduction was much lower or not present under heat and drought stress. Moreover, the starch content of the tubers was hardly affected by stress treatments. Together, we provide a novel strategy to adopt potato plants to withstand expected climate changes and help to secure future carbohydrate food production while saving water resources at the same time.

## Results

### Simultaneous Expression of Hexokinase and SP6A in Transgenic Potato Plants Reduces Transpiration and Enhances Tuberization

In previous studies it has been shown that guard cell-specific expression of *AtHXK1* improves WUE of several plant species (Kelly et al., 2013, 2017; Lugassi et al., 2015). To verify that guard cell-specific expression of *AtHXK1* in potato leads to reduced stomatal conductance and transpiration rates, transgenic potato lines overexpressing *AtHXK1* under the guard cell-specific KST1 promoter (GCHXK) were created. Transgene expression was verified by qPCR in two independent lines (Supplementary Figure S1A). Next, gas exchange parameters were measured in these transgenic lines which confirmed reduced stomatal conductance (Supplementary Figure S1B) and transpiration rates (Supplementary Figure S1C). Concurrently, no clear negative effect on CO_2_ assimilation (Supplementary Figure S1D) was detected in these lines resulting in an enhanced WUE (Supplementary Figure S1E). Additionally, tuber yield of the highest expressing line was increased which can be attributed to a higher tuber weight rather than an increased tuber number (Supplementary Figures S1H,G).

**FIGURE 1:**
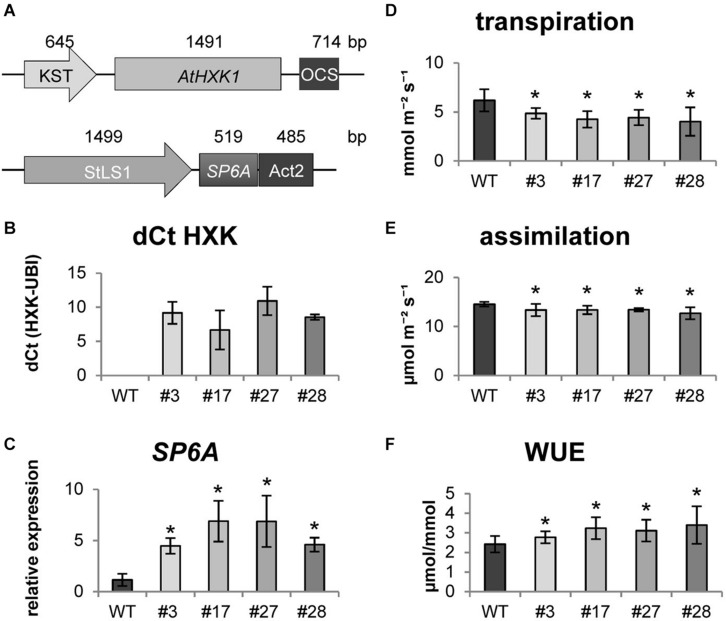
Characterisation of HXK+SP6A potato plants under ambient conditions **(A)** schematic depiction of gene constructs **(B)** expression of AtHXK1 in source leaves, **(C)** expression of *SP6A* in source leaves, **(D)** transpiration **(E)** assimilation, **(F)** water use efficiency (WUE) at 28 days; values are the mean of four biological replicates ± SD, *p ≤ 0.05.

Next, we attempted to co-express both, *AtHXK1* and *SP6A*, in transgenic potato plants. Therefore both constructs were assembled into one expression vector using the Golden Gate cloning system (Weber et al., 2011; Figure 1A). *AtHXK1* was expressed under the KST1 promoter (Müller-Röber et al., 1995), whereas *SP6A* was expressed under the StLS1 promoter (Stockhaus et al., 1987). Four transgenic lines were selected expressing both genes at high levels in source leaves (Figures 1B,C). Compared to untransformed control plants all transgenic plants showed a bushy habitus with reduced plant height (Supplementary Figure S2A). To investigate this further, several morphological parameters were measured (Supplementary Figures S2B–D), which indicate that the lower shoot length was accompanied by a higher number of leaves. Even though the individual leaves were smaller, the total leaf area per plant was slightly, but significantly, increased in all transgenic lines.

Following the molecular and morphological characterization, we investigated physiological changes in AtHXK1+SP6A plants. As shown in Figure 1D, transpiration rate of the double transgenic lines was reduced compared to the wild type. The lower transpiration was most likely caused by less opened stomata due to *AtHXK1* expression which was confirmed by measuring the stomata width and length (Supplementary Figure S3A). The ratio between both parameters was significantly reduced in the transgenic lines, while the number of stomata per leaf area was similar to the wild type (Supplementary Figure S3B). Thus, the less opened stomata led to an about 30% lower transpiration, but at the same time the CO_2_ assimilation was only slightly negatively influenced (reduction by 10%) (Figure 1E) leading to an approximately 30% increased WUE in the transgenic lines (Figure 1F).

### Transgenic AtHXK1+SP6A Potato Plants Show High Yield Stability Under Heat and Drought Stress

**FIGURE 2:**
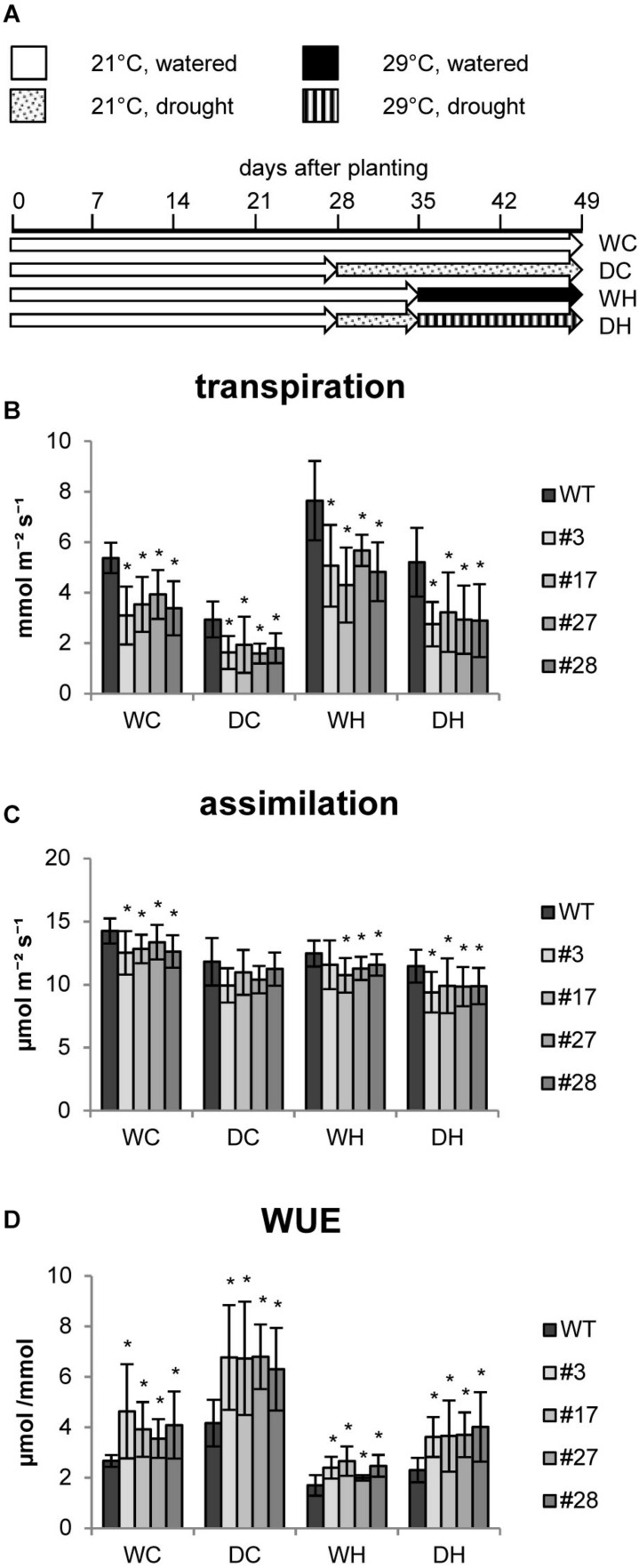
Photosynthesis of HXK+SP6A potato plants under different stress treatments at the age of 5–6 weeks. **(A)** Experimental setup, **(B)** transpiration, **(C)** assimliation, **(D)** WUE, values are the mean of 8–12 biological replicates ± SD, *p ≤ 0.05 compared to respective WT.

In further studies we investigated whether these transgenic plants are more resilient to abiotic stress factors and examined physiological and biochemical responses to heat and drought stress and a combination thereof. We designed an experimental setup (Figure 2A) in which all plants were first grown for 4 weeks under well-watered control conditions (approx. 65% relative water content (RWC) in the soil). Then, half of the population was adapted to drought conditions (approx. 35% RWC) for 1 week. Soil humidity was adjusted by daily watering. Thereafter, one half of each group was shifted to elevated temperatures in order to investigate heat effects. All plants were grown for two more weeks under these conditions until harvest (Figure 2A).

The transpiration rate of the plants was determined under each condition. Similar to the first experiments, the transpiration rates were reduced in all AtHXK1+SP6A lines as compared to wild-type plants grown under control conditions (WC = water control) (Figure 2B). The transpiration rates of both wild type and AtHXK1+SP6A lines decreased in response to drought (DC = drought control), but total evaporation was lower in the transgenic plants (Figure 2B) consistent with the reduced stomatal aperture. Under elevated temperatures (WH = water heat), wild-type plants increased transpiration. A similar response was seen in AtHXK1+SP6A lines, but the absolute values were about 35% less than in wild-type plants (Figure 2B). Under combined stress conditions, i.e., drought and heat (DH), again the transpiration rate of transgenic lines was lower compared to wild-type plants.

CO_2_ assimilation was, if at all, only mildly reduced in AtHXK1+SP6A lines under all growth conditions as compared to wild-type plants (Figure 2C). Consequently, WUE was improved by about 30–50% in the AtHXK1+SP6A lines under well-watered control (WC), drought (DC), heat (WH) as well as double stress (DH) conditions (Figure 2D). This effect was mainly driven by the lower transpiration rate caused by expression of AtHXK1.

**FIGURE 3:**
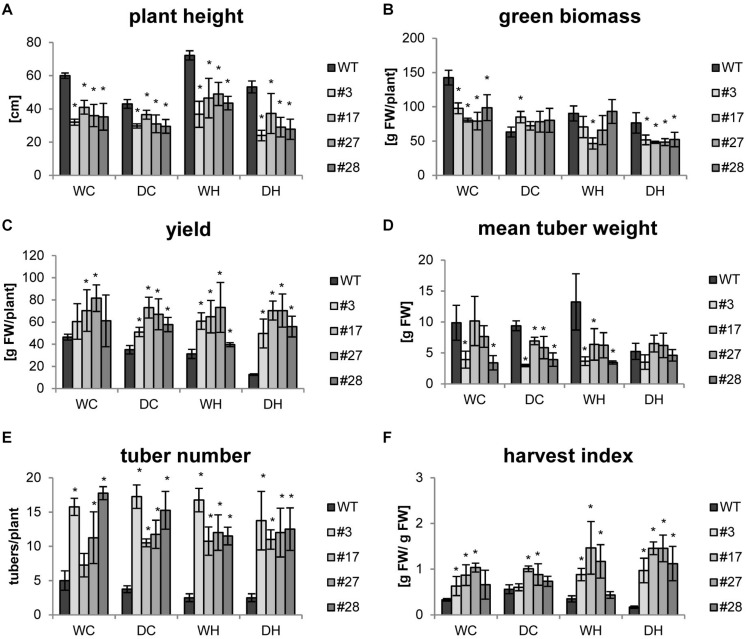
Yields data for HXK+SP6A potato plants harvested at 49 days. **(A)** Plant height, **(B)** green biomass, **(C)** yield, **(D)** mean tuber weight, **(E)** tuber number, **(F)** harvest index; plants were harvested at the age of 7 weeks; values are the mean of 4 plants ± SD, *p ≤ 0.05 compared to respective WT.

As the focus of our research was a biotechnological improvement of the tuber crop potato, we were especially interested in tuber yield which was determined together with other growth parameters at the end of the experiments. As observed before, all transgenic plants exhibited a reduced shoot growth compared to wild-type plants under control conditions that persisted also under all stress treatments (Figure 3A). Under drought conditions, plant height as well as green biomass (e.g., leaves and stem) decreased in the wild type, while both parameters were less affected in the transgenic lines (Figures 3A,B). The increase in plant height under heat (WH), known as shade avoidance phenotype, was observed in all genotypes, but it was less pronounced in the transgenics. The heat-mediated shoot elongation was abolished by simultaneous drought and heat stress (DH) in wild-type plants. Under combined stress the transgenic lines were slightly smaller than their respective controls (Figure 3A). The green biomass accumulation seemed less impaired by drought stress in the transgenics compared to wild type. Most importantly, despite a lower green biomass, tuber yield of all transgenic lines was 30–70% higher under control conditions. Tuber yield of transgenic plants was hardly affected by stress applications, indicating that they maintain high tuber yields even under stress conditions (Figure 3C and Supplementary Figure S4). In contrast, double stress application (DH) led to a severe yield reduction of roughly 70% in wild-type plants (Figure 3C). Under these conditions, the overall tuber yield of the transgenic plants was 4–5 times higher as compared to wild-type plants (Figure 3C). Even though tubers of transgenic plants were smaller (Figure 3D), a massive increase in tuber number more than compensated for this (Figure 3E) and contributed to higher yields. Finally, the harvest index of the transgenic plants was 2–8 times higher under all conditions (Figure 3F). The positive effects of AtHXK1 and SP6A co-expression especially on tuber yield were observed in three independent experiments using four plants per treatment and independent transgenic lines underlining the high reproducibility (Figure 3 and Supplementary Figures S5, S6).

As a most likely reason for the yield reduction in the wild type, we reasoned a decreased *SP6A* expression in leaves and performed a quantitative RT-PCR. In fact, *SP6A* expression decreased upon both drought and heat in the wild type (Supplementary Figure S7A). However, the strongest downregulation of *SP6A* was detected under combined stress conditions. Overall, the pattern of yield reduction fitted well with *SP6A* expression level (Pearson correlation 0.91). In contrast, higher *SP6A* mRNA levels were measured in all transgenic lines under all conditions (Supplementary Figure S7A). In addition, expression levels of the small RNA (*SES*) described previously to repress *SP6A* under heat (Lehretz et al., 2019), was measured in wild type. The results confirmed its heat-mediated induction in wild type, while drought stress resulted in a down-regulation of *SES* (Supplementary Figure S7B).

**FIGURE 4:**
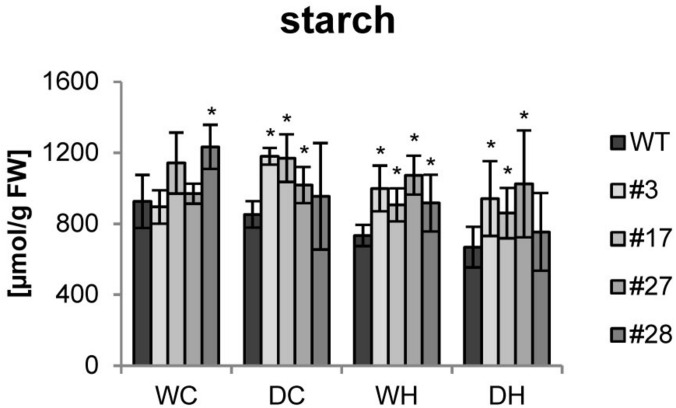
Starch content of tubers of HXK+SP6A potato plants under different stress treatments. Bars show mean of four biological replicates ± SD, *p ≤ 0.05 compared to respective WT.

Since more tubers per plant would have no agricultural value with reduced dry matter, we measured the tuber starch content. Under control conditions, no clear changes between tubers from wild type or transgenic plants were detected. Upon drought stress, no significant reduction in the starch content was seen in wild-type tubers. However, starch levels clearly decreased in wild type in response to heat and even further by combined heat and drought stress (Figure 4). Remarkably, starch contents were not significantly altered by stress treatments in most of the transgenic tubers and higher amounts of starch than in the corresponding wild-type tubers were observed under stress conditions (Figure 4).

## Discussion

Since global average temperatures are expected to rise further in the near future designing crop plants which can withstand heat stress is of utmost importance to secure food production (Birch et al., 2012; George et al., 2017; Lamaoui et al., 2018). Additionally, heat is often accompanied by drought, especially in tepid climate. Therefore, reduction of transpiration is a very desirable trait as it stabilizes yield and unleashes resources, allowing to be used elsewhere. With this goal in mind, transgenic potato plants were created that co-express two target genes, *AtHXK1* and *SP6A*, and were tested for improved stress tolerance toward heat, drought and combined stress conditions.

The water consumption of crop plants is an important topic. Even though potato is rather efficient in water usage compared to other crops it is still vulnerable to drought conditions. Decreased transpiration rates might solve this agricultural problem. AtHXK1 was shown to reduce transpiration when expressed in guard cells of *A. thaliana*, citrus and tomato without a negative effect on plant growth and CO_2_ assimilation (Kelly et al., 2013, 2019; Lugassi et al., 2015). Although the molecular mechanism is not completely understood, it is assumed that AtHXK1 controls stomatal aperture to coordinate photosynthesis with transpiration through sugar signaling pathways (Kottapalli et al., 2018; Granot and Kelly, 2019) as it has been described as a sugar sensor before (Moore et al., 2003). According to a current model, it is supposed that a surplus of sucrose that is not transported by the phloem during high photosynthetic activity is carried with the transpiration stream toward the guard cells where it serves as a signal to close the stomata and thereby prevents unnecessary water loss (Kelly et al., 2013; Lugassi et al., 2015; Kottapalli et al., 2018).

We expressed the KST::AtHXK1 construct alone and together with *SP6A* in potato and provided clear evidence that the desired effect of *AtHXK1* expression (e.g., reduced transpiration) is also present in this crop plant under control conditions as shown for Arabidopsis (Kelly et al., 2013), citrus (Lugassi et al., 2015) and tomato (Kelly et al., 2019). Concurrently, stomata-specific expression of *AtHXK1* slightly affected photosynthetic CO_2_ assimilation in potato. In concert, both effects led to a significantly increased water use efficiency. Although CO_2_ assimilation per leaf area was negatively affected in the HXK+SP6A plants, the bushy growth phenotype, caused by *SP6A* overexpression, with more (smaller) leaves might counteract for this.

One common response to drought is stomatal closure (Liu et al., 2006). Therefore, we reasoned that plants with already lower stomatal conductance may have an advantage under limited water availability. Accordingly, transpiration decreased under drought stress in wild-type plants as well as in AtHXK1+SP6A lines with the transgenic lines exhibiting a lower transpiration rate as compared to wild-type plants. The same effect was seen in heat and drought combination, while heat alone resulted overall in increased transpiration rates as described before (Hancock et al., 2014; Hastilestari et al., 2018). Thus, we conclude that reduced stomatal conductance helps to safe water under drought stress, but a lower transpiration is also advantageous under mild heat stress and combined heat and drought stress.

In order to stimulate tuberization the *FLOWERING LOCUS T* homologue *SP6A* was selected for overexpression. This gene was identified as a positive key regulator of tuberization that is controlled by the photoperiod pathway (Navarro et al., 2011). Hence, overexpression of *SP6A* in potato promoted tuberization in the short day-dependent *S. andigena* in a day length independent manner (Navarro et al., 2011) and also in modern day-length insensitive cultivars (Teo et al., 2017). We recently showed that overexpression of a codon optimized *SP6A* version in the tetraploid variety Solara was accompanied by a severely altered source-sink balance (Lehretz et al., 2019).

Furthermore, the downregulation in *SP6A* expression is seen as a cause for the heat-mediated inhibition of tuber formation (Hancock et al., 2014; Hastilestari et al., 2018). This occurs via transcriptional and post-transcriptional regulation. A small RNA was identified which targets the *SP6A* transcript for post-transcriptional degradation. Importantly, the expression of this small RNA was strongly induced under elevated temperatures and using a target mimicry approach the functional relevance of the small RNA for the regulation of *SP6A* under heat was demonstrated (Lehretz et al., 2019).

Our approach described here led to elevated *SP6A* levels and stimulated tuber formation under control conditions. By using the StLS1 promoter *SP6A* levels were only moderately increased (five to sevenfold) in chloroplast-containing cells and consequently the development of the shoot was not as strongly affected as compared to our previous approach using a codon-optimized *SP6A* transcript driven by the constitutive CaMV 35S promoter. Nevertheless, AtHXK1+SP6A overexpressing plants were smaller and accumulated less aboveground biomass than wild-type plants, but tuber number and yield increased significantly.

Under elevated temperatures as well as under drought stress tuber number and yield of wild-type potato plants were significantly decreased as reported in previous studies (Dahal et al., 2019). The heat-mediated decline in tuber yield is associated with a strong decrease in *SP6A* expression. For drought, this correlation has not been shown yet. In our study, we found that *SP6A* expression is inhibited by drought and most significantly by a combination of drought and heat stress. The strong correlation between *SP6A* expression and yield further supports the idea that *SP6A* is an important target for ensuring stable yields under changing environmental conditions.

Under heat conditions, the small RNA *SES* inhibits *SP6A* expression and is responsible for the observed yield decrease. Under drought conditions, we did not find increased *SES* expression levels. Therefore, we conclude that *SES* is not responsible for downregulation of *SP6A* under drought stress conditions. Instead, other mechanisms, such as posttranslational repression of members in the *CDF/CO* pathway or other miRNAs will be responsible for the observed *SP6A* regulation. Further studies are needed to unravel the molecular mechanisms responsible for the observed drought-mediated downregulation of *SP6A* expression. Taken together, high *SP6A* expression levels under conditions of water scarcity and elevated temperatures will be crucial to maintain high tuber yield under conditions of expected climate change.

Tuber fresh weight and tuber number of the transgenic HXK+SP6A lines were only slightly affected by the applied stress conditions. Even though *SP6A* transcript levels decreased in some transgenic lines under stress conditions, the remaining transcript level was three to fourfold higher compared to wild type under control conditions and therefore most likely sufficient to maintain tuberization and to further support assimilate translocation into growing tubers. SP6A was recently shown to interact with the sucrose efflux carrier SWEET11 and thereby prevents leakage of sucrose into the apoplast. It is assumed that this enhances assimilate allocation and promotes symplasmic unloading of sucrose into developing tubers (Abelenda et al., 2019). During tuber development the mode of sucrose unloading switches from apoplasmic to symplasmic (Viola et al., 2007). Thus, the interaction of SP6A with SWEET11 provides a functional link between photoperiodic control and assimilate unloading into developing tubers.

Consistent with this hypothesis, the starch content of the transgenic tubers was largely unaffected, under single and double stress, while it decreased with the severity of stress in the wild type. Together these results further support the assumption that SP6A plays a major role in development of potato tubers and maintenance of their sink strength.

Together, AtHXK1+SP6A co-expression combines a sustainable use of limited water resources with a high tuberization capacity under greenhouse conditions and thereby helps to maintain tuber formation and growth under heat, drought and combined stress conditions. Whether this positive effect is maintained under open field conditions and whether improved abiotic stress tolerance might negatively interfere with biotic stress responses needs to be validated in further studies.

## Materials and Methods

### Plant Material and Growth Conditions

Potato (*Solanum tuberosum* L. cv. Désirée) was used for the experiments with the GCHXK lines. These plants were grown in a mixture of peat, quartz, and coconut fibers (Green 90, Even Ari, Israel) in a temperature-controlled greenhouse under natural conditions.

For all other experiments, *S. tuberosum L.* cv. Solara plants were grown under greenhouse conditions in 3.5 l pots (20 cm diameter) with 16 h supplemental lights (250 μE). Plants were maintained and amplified in tissue culture on Murashige and Skoog medium (Murashige and Skoog, 1962) containing 2% sucrose. Walk-in growth chambers were used for controlled ambient and elevated temperature treatments (22 or 29°C during light period and 20 or 27°C during dark period, respectively) with supplemental light (350 μE). Drought stress was applied by stopping watering for 2–3 days and subsequent adjusting the relative water content (RWC) in the soil to 35%, while in control condition RWC was 65%. This was achieved through measurement of soil conductivity using the EM50 soil moisture sensor (Decagon, United States) and calibration of pot weight. Leaf area was measured using a LI-3100 area meter (LI-COR, United States). Plant height was measured from soil surface to apical meristem. Harvest index was calculated as ratio of tuber fresh weight per plant over green fresh weight per plant.

### Plasmid Construction and Generation of Transgenic Plants

The GCHXK lines were generated by transforming the KST*pro*::AtHXK1 constructs described previously (Kelly et al., 2013) in potato plants (cv. Désirée) using the *Agrobacterium tumefaciens* strain EHA105. Following the screening on Kanamycin selection media, PCR with KST specific primers was used to distinguish between transgenic and non-transgenic plants. Twenty positive plants were identified. Positive plants were then checked for their gas exchange parameters, and two lines with significant reduction in transpiration, GCHXK3 and GCHXK12 were selected for further analysis.

The stacked construct was generated using the GoldenGate cloning system (Engler et al., 2008, 2009; Weber et al., 2011). Synthetic or PCR amplified gene sequences (Thermo Fisher Scientific GeneArt GmbH) and modules available from MoClo plant part kits^1^ (Werner et al., 2012) were assembled into level 0 and subsequently level 1 vectors to generate the plasmids St-LS1::*StSP6A* and *KST1::AtHXK1*. Both plasmids were combined with a kanamycin-resistance cassette (pICSL70004) into a level 2 vector (pAGM4237, Addgene) which was transformed into potato cv. Solara by *Agrobacterium*-mediated gene transfer to generate the AtHXK1+SP6A lines (Rocha-Sosa et al., 1989). An overview of modules used to generate the plasmid is provided in Supplementary Table S2. Thirty-four transgenic lines were obtained and screened for the presence of both genes by qPCR. Four lines (# 3, 17, 27, 28) were selected for further studies that express both targets.

### RNA Isolation, cDNA Synthesis, and Quantitative RT-PCR Analysis

Samples of source leaves were taken during the first half of the light period, 4 h after dawn. Total RNA was isolated from ca. 100 mg of frozen leaf material by grinding on 8M guanidiumchloride and 0.7% β-ME (Logemann et al., 1987). RNA quantity was measured with ND-1000 Spectrophotometer (NanoDrop Technologies). Complementary DNA synthesis and quantitative real time PCR (qPCR) analyses were conducted as described previously (Ferreira and Sonnewald, 2012). Quantitative Real-Time PCR (qPCR) was conducted on AriaMx (Agilent Technologies) and analyzed as described by Livak and Schmittgen (2001). Alternatively, RNA extraction, cDNA preparation, and quantitative real-time PCR analysis were performed as described before (Lugassi et al., 2015). Data were normalized using NAC (XM_006339185) or Ubi 3 (L22576) as reference genes. The primers used for amplification are listed in Supplementary Table S1.

### Sugar Measurements

Starch was extracted using ca. 50 mg of tuber FW grinded in 80% Ethanol, incubated with 0.2 M KOH overnight, heated at 95°C for 90 min and neutralized with 1 N acidic acid. After amyloglucosidase digestion glucose contents were determined using a coupled optical assay as described previously (Hastilestari et al., 2018).

### Photosynthesis Measurements

Photosynthesis, transpiration rates and stomatal conductance were measured on fully developed source leaves on the upper middle stem (5th–8th from top) under greenhouse conditions using a LI-COR 6800 or LI-COR 6400 device. All measurements were conducted between 3 and 6 h after dawn (09:00–12:00 a.m.) under the respective greenhouse conditions (400–600 μmol m^–2^ s^–2^ light, 400–500 μmol mol^–1^ CO_2_, 50% relative humidity). WUE was calculated as assimilation/transpiration. Temperature was also adjusted according to the treatment in the greenhouse (22 and 29°C, respectively).

### Microscopic Analysis of Stomata

In order to measure the length to width ratio of stomata source leaves were coated with transparent quick dry nail polish by brushing the abaxial side of fully developed source leaves 4 h after dawn. The coatings were immediately removed after drying and photographed with a Leica DMR Microscope. Lengths were measured using the FIJI software.

### Statistical Analysis

Statistical analyses were done by Student’s *t*-test. Specific details of the statistical test used, number of biological and technical replicates, and the description of error bars are included in the figure legends.

## Data Availability Statement

The original contributions presented in the study are included in the article/Supplementary Material, further inquiries can be directed to the corresponding author/s.

## Author Contributions

NL and DG designed and tested GCHXK lines. GL designed and performed all other experiments. GL and SS analyzed data. SS and US were responsible for project planning. GL, SS, and US wrote the manuscript with input from all other authors. All authors contributed to the article and approved the submitted version.

## Conflict of Interest

The authors declare that the research was conducted in the absence of any commercial or financial relationships that could be construed as a potential conflict of interest.

## Footnotes

1 http://www.addgene.org

## Supplementary Material

Supplementary Table 1 Primer sequences.

Supplementary Table 2 AtHXK1+SP6A construct assembly.

## References

[B1] Abelenda J. A. Bergonzi S. Oortwijn M. Sonnewald S. Du M. Visser R. (2019). ‘Source-sink regulation is mediated by interaction of an FT- homolog with a SWEET protein in potato’. *Curr. Biol.* 29 1178–1186. 10.1016/j.cub.2019.02.018 30905604

[B2] Abelenda J. A. Navarro C. Prat S. (2011). ‘From the model to the crop: Genes controlling tuber formation in potato’. *Curr. Opin. Biotechnol.* 22 287–292. 10.1016/j.copbio.2010.11.013 21168321

[B3] Abelenda J. A. Navarro C. Prat S. (2014). ‘Flowering and tuberization: A tale of two nightshades’. *Trends Plant Sci.* 19 115–122. 10.1016/j.tplants.2013.09.010 24139978

[B4] Birch P. R. J. Bryan G. Fenton B. Gilroay E. M. Hein I. Jones J. T. (2012). Crops that feed the world 8: Potato: Are the trends of increased global production sustainable? *Food Secur.* 4 477–508. 10.1007/s12571-012-0220-1

[B5] Dahal K. Li X. Q. Tai H. Creelman A. Bizimungu B. (2019). ‘Improving potato stress tolerance and tuber yield under a climate change scenario – a current overview’. *Front. Plant Sci.* 10 1–16. 10.3389/fpls.2019.00563 31139199 PMC6527881

[B6] Deblonde P. M. K. Ledent J. F. (2001). ‘Effects of moderate drought conditions on green leaf number, stem height, leaf length and tuber yield of potato cultivars’. *Eur. J. Agron.* 14 31–41. 10.1016/s1161-0301(00)00081-2

[B7] Engler C. Gruetzner R. Kandzia R. Marillonnet S. (2009). ‘Golden gate shuffling: A one-pot DNA shuffling method based on type ils restriction enzymes’. *PLoS One* 4:e5553. 10.1371/journal.pone.0005553 19436741 PMC2677662

[B8] Engler C. Kandzia R. Marillonnet S. (2008). A one pot, one step, precision cloning method with high throughput capability’. *PLoS One* 3:e3647. 10.1371/journal.pone.0003647 18985154 PMC2574415

[B9] Fahad S. Bajwa A. A. Nazir U. Anjum S. A. Farooq A. Zohaib A. (2017). Crop Production under Drought and Heat Stress: Plant Responses and Management Options. *Front. Plant Sci.* 8:1147. 10.3389/fpls.2017.01147 28706531 PMC5489704

[B10] Ferreira S. J. Sonnewald U. (2012). ‘The mode of sucrose degradation in potato tubers determines the fate of assimilate utilization’. *Front. Plant Sci.* 3:23. 10.3389/fpls.2012.00023 22639642 PMC3355675

[B11] George T. S. Taylor M. A. Dodd I. C. White P. J. (2017). (2017) ‘Climate Change and Consequences for Potato Production: a Review of Tolerance to Emerging Abiotic Stress’. *Potato Res.* 60 239–268. 10.1007/s11540-018-9366-3

[B12] Granot D. Kelly G. (2019). ‘Evolution of Guard-Cell Theories: The Story of Sugars’. *Trends Plant Sci.* 24 507–518. 10.1016/j.tplants.2019.02.009 30862392

[B13] Hancock R. D. Morris W. L. Ducreux L. J. M. Morris J. A. Usman M. Verrall S. R. (2014). ‘Physiological, biochemical and molecular responses of the potato (Solanum tuberosumL.) plant to moderately elevated temperature’. *Plant Cell Environ.* 37 439–450. 10.1111/pce.12168 23889235

[B14] Hannapel D. J. Sharma P. Lin T. Banerjee A. K. (2017). ‘The Multiple Signals That Control Tuber Formation’. *Plant Physiol.* 174 845–856. 10.1104/pp.17.00272 28520554 PMC5462066

[B15] Hastilestari B. R. Lorenz J. Reid S. Hofmann J. Pscheidt D. Sonnewald S. (2018). ‘Deciphering source and sink responses of potato plants (Solanum tuberosum L.) to elevated temperatures’. *Plant Cell Environ.* 41 2600–2616. 10.1111/pce.13366 29869794

[B16] Kelly G. Egbaria A. Khamaisi B. Lugassi N. Attia Z. Moshelion M. (2019). ‘Guard-Cell Hexokinase Increases Water-Use Efficiency Under Normal and Drought Conditions’. *Front. Plant Sci.* 10:1499. 10.3389/fpls.2019.01499 31803219 PMC6877735

[B17] Kelly G. Lugassi B. E. Wolf D. Khamaisi B. Brandsma D. (2017). The Solanum tuberosum KST1 partial promoter as a tool for guard cell expression in multiple plant species’. *J. Exp. Bot.* 68 2885–2897. 10.1093/jxb/erx159 28531314 PMC5853950

[B18] Kelly G. Moshelion M. David-Schwartz R. Halperin O. Wallach R. Attia Z. (2013). ‘Hexokinase mediates stomatal closure’. *Plant J.* 75 977–988. 10.1111/tpj.12258 23738737

[B19] Kottapalli J. David-Schwartz R. Khamaisi B. Brandsma D. Lugassi N. Egbaria A. (2018). ‘Sucrose-induced stomatal closure is conserved across evolution’. *PLoS One* 13:e020535. 10.1371/journal.pone.0205359 30312346 PMC6185732

[B20] Lafta A. M. Lorenzen J. H. (1995). ‘Effect of High Temperature on Plant Grwoth and Carbohydrate Metabolism in Potato Plants’. *J. Plant Physiol.* 109 637–643. 10.1104/pp.109.2.637 12228617 PMC157630

[B21] Lamaoui M. Jemo M. Datla R. Bekkaoui F. (2018). ‘Heat and drought stresses in crops and approaches for their mitigation’. *Front. Chem.* 6:26. 10.3389/fchem.2018.00026 29520357 PMC5827537

[B22] Lehretz G. G. Sonnewald S. Hornyik C. Corral J. M. Sonnewald U. (2019). ‘Post-transcriptional Regulation of FLOWERING LOCUS T Modulates Heat-Dependent Source-Sink Development in Potato’. *Curr. Biol.* 29 1614–1624. 10.1016/j.cub.2019.04.027 31056391

[B23] Levy D. Veilleux R. E. (2007). ‘Adaptation of potato to high temperatures and salinity - A review’. *Am. J. Potato Res.* 84 487–506. 10.1007/bf02987885

[B24] Liu F. Shahnazari A. Andersen M. Jacobsen S. E. Jensen C. R. (2006). ‘Physiological responses of potato (Solanum tuberosum L.) to partial root-zone drying: ABA signalling, leaf gas exchange, and water use efficiency’. *J. Exp. Bot.* 57 3727–3735. 10.1093/jxb/erl131 16982651

[B25] Livak K. J. Schmittgen T. D. (2001). ‘Analysis of relative gene expression data using real-time quantitative PCR and the 2-ΔΔCT method’. *Methods* 25 402–408. 10.1006/meth.2001.1262 11846609

[B26] Logemann J. Schell J. Willmitzer L. (1987). ‘Improved Method for the Isolation of RNA from Plant Tissues’. *Analyt. Biochem.* 163 16–20. 10.1016/0003-2697(87)90086-8 2441623

[B27] Lugassi N. Kelly G. Fidel L. Yaniv Y. Attia Z. Levi A. (2015). ‘Expression of Arabidopsis Hexokinase in Citrus Guard Cells Controls Stomatal Aperture and Reduces Transpiration’. *Front. Plant Sci.* 6:1114. 10.3389/fpls.2015.01114 26734024 PMC4679854

[B28] Mittler R. Finka A. Goloubinoff P. (2012). ‘How do plants feel the heat?’. *Trends Biochem. Sci.* 37 118–125. 10.1016/j.tibs.2011.11.007 22236506

[B29] Monneveux P. Ramírez D. A. Pinob M.-T. (2013). ‘Drought tolerance in potato (S. tuberosum L.): Can we learn from drought tolerance research in cereals?’. *Plant Sci.* 205 76–86. 10.1016/j.plantsci.2013.01.011 23498865

[B30] Moon J. Han S. Kim D. Yoon I. Shin D. Byun M. (2015). ‘Ectopic expression of a hot pepper bZIP - like transcription factor in potato enhances drought tolerance without decreasing tuber yield’. *Plant Mole. Biol.* 89 421–431. 10.1007/s11103-015-0378-y 26394867

[B31] Moore B. Zhou L. Rolland F. Hall Q. Cheng W. Liu Y. (2003). ‘Role of the Arabidopsis glucose sensor HXK1 in nutrient, light, and hormonal signaling’. *Science* 300 332–336. 10.1126/science.1080585 12690200

[B32] Morris W. L. Ducreux L. J. M. Morris J. Campbell R. Usman M. Hedley P. E. (2019). Identification of TIMING of CAB EXPRESSION 1 as a temperature-sensitive negative regulator of tuberization in potato’. *J. Exp. Bot.* 70 5703–5714. 10.1093/jxb/erz336 31328229 PMC6812706

[B33] Müller-Röber B. Ellenberg J. Provart N. Willmitzer L. Busch H. Becker D. (1995). ‘Cloning and electrophysiological analysis of KST1’. *EMBO J.* 14 2409–2416. 10.1002/j.1460-2075.1995.tb07238.x 7781596 PMC398354

[B34] Murashige T. Skoog S. (1962). A revised medium for rapid growth and bio assays with tobacco tissue cultures. *Physiologia Plantarum* 15, 473–497. 10.1111/j.1399-3054.1962.tb08052.x

[B35] Navarro C. Abelenda J. A. Cruz-Oró E. Cuéllar C. A. Tamaki S. Silva J. (2011). Control of flowering and storage organ formation in potato by FLOWERING LOCUS T’. *Nature* 478 119–122. 10.1038/nature10431 21947007

[B36] Navarro C. Cruz-Oró E. Prat S. (2015). ‘Conserved function of FLOWERING LOCUS T (FT) homologues as signals for storage organ differentiation’. *Curr. Opin. Plant Biol.* 23 45–53. 10.1016/j.pbi.2014.10.008 25449726

[B37] Rocha-Sosa M. Sonnewald U. Frommer W. Stratmann M. Schell J. Willmitzer L. (1989). ‘Both developmental and metabolic signals activate the promoter of a class I patatin gene’. *EMBO J.* 8 23–29. 10.1002/j.1460-2075.1989.tb03344.x 16453867 PMC400768

[B38] Schafleitner R. Gutierrez-Rosales R. O. Gaudin A. Alvarado Aliaga A. A. Nomberto Martinez G. Tincopa Marca L. R. (2007). Capturing candidate drought tolerance traits in two native Andean potato clones by transcription profiling of field grown plants under water stress’. *Plant Physiol. Biochem.* 45 673–690. 10.1016/j.plaphy.2007.06.003 17764965

[B39] Shin D. Moon S. Han S. Kim B. Park S. R. Lee S. (2011). ‘Expression of StMYB1R-1, a novel potato single MYB-like domain transcription factor, increases drought tolerance’. *Plant Physiol.* 155 421–432. 10.1104/pp.110.163634 21030505 PMC3075785

[B40] Stockhaus J. Eckes P. Rocha-Sosa M. Schell J. Willmitzer L. (1987). ‘Analysis of cis-active sequences involved in the leaf-specific expression of a potato gene in transgenic plants’. *Proc. Natl. Acad. Sci.* 84 7943–7947. 10.1073/pnas.84.22.7943 16593893 PMC299452

[B41] Teo C. J. Takahashi K. Shimizu K. Shimamoto K. Taoka K. I. (2017). ‘Potato tuber induction is regulated by interactions between components of a tuberigen complex’. *Plant Cell Physiol.* 58 365–374. 28028166 10.1093/pcp/pcw197

[B42] Trapero-Mozos A. Ducreux L. J. M. Bita C. E. Morris W. Wise C. Morris J. A. (2018). ‘A reversible light - and genotype - dependent acquired thermotolerance response protects the potato plant from damage due to excessive temperature’. *Planta* 247 1377–1392. 10.1007/s00425-018-2874-1 29520461 PMC5945765

[B43] Trapero-Mozos A. Morris W. L. Ducreux L. J. M. McLean K. Stephens J. Torrance L. (2018). ‘Engineering heat tolerance in potato by temperature-dependent expression of a specific allele of HEAT-SHOCK COGNATE 70’. *Plant Biotechnol. J.* 16 197–207. 10.1111/pbi.12760 28509353 PMC5785350

[B44] Viola R. Pelloux J. van der Ploeg A. Gillespie T. Marquis N. Roberts A. R. (2007). ‘Symplastic connection is required for bud outgrowth following dormancy in potato (Solanum tuberosum L.) tubers’. *Plant Cell Environ.* 30 973–983. 10.1111/j.1365-3040.2007.01692.x 17617825

[B45] Weber E. Engler C. Gruetzner R. Werner S. Marillonnet S. (2011). ‘A modular cloning system for standardized assembly of multigene constructs’. *PLoS One* 6:e16765. 10.1371/journal.pone.0016765 21364738 PMC3041749

[B46] Werner S. Engler C. Weber E. Gruetzner R. Marillonnet S. (2012). ‘Fast track assembly of multigene constructs using golden gate cloning and the MoClo system’. *Bioengin. Bugs* 3 38–43. 10.4161/bbug.3.1.18223 22126803

