# Supplementary figures and images for: Future-Proofing Potato for Drought and Heat Tolerance by Overexpression of Hexokinase and SP6A
Source: Front Plant Sci. 2021 Jan 12;11:614534. doi: 10.3389/fpls.2020.614534 (PMC7835534; doi:10.3389/fpls.2020.614534)

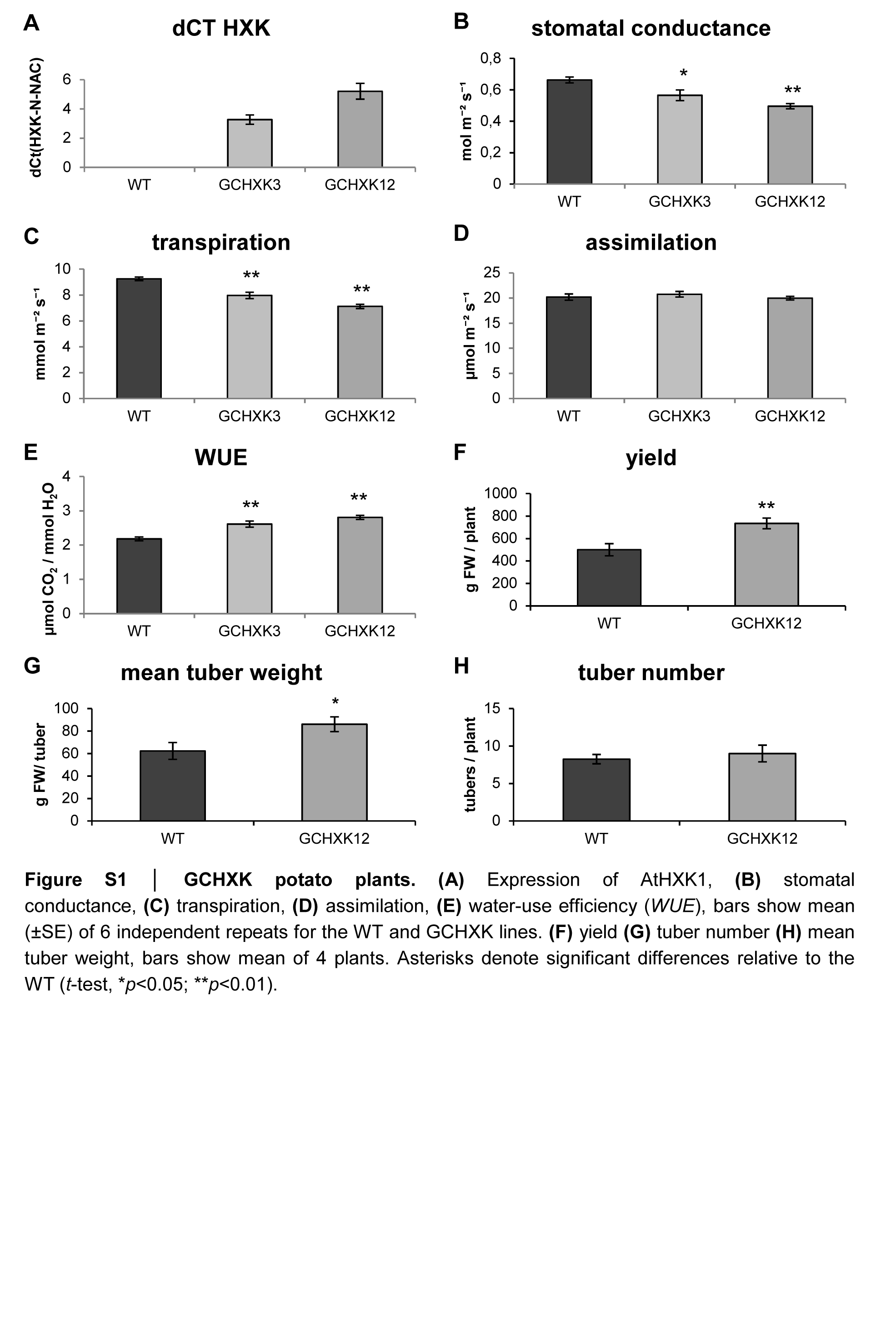

Supplement: Supplementary file 3 [file Image_1.png]

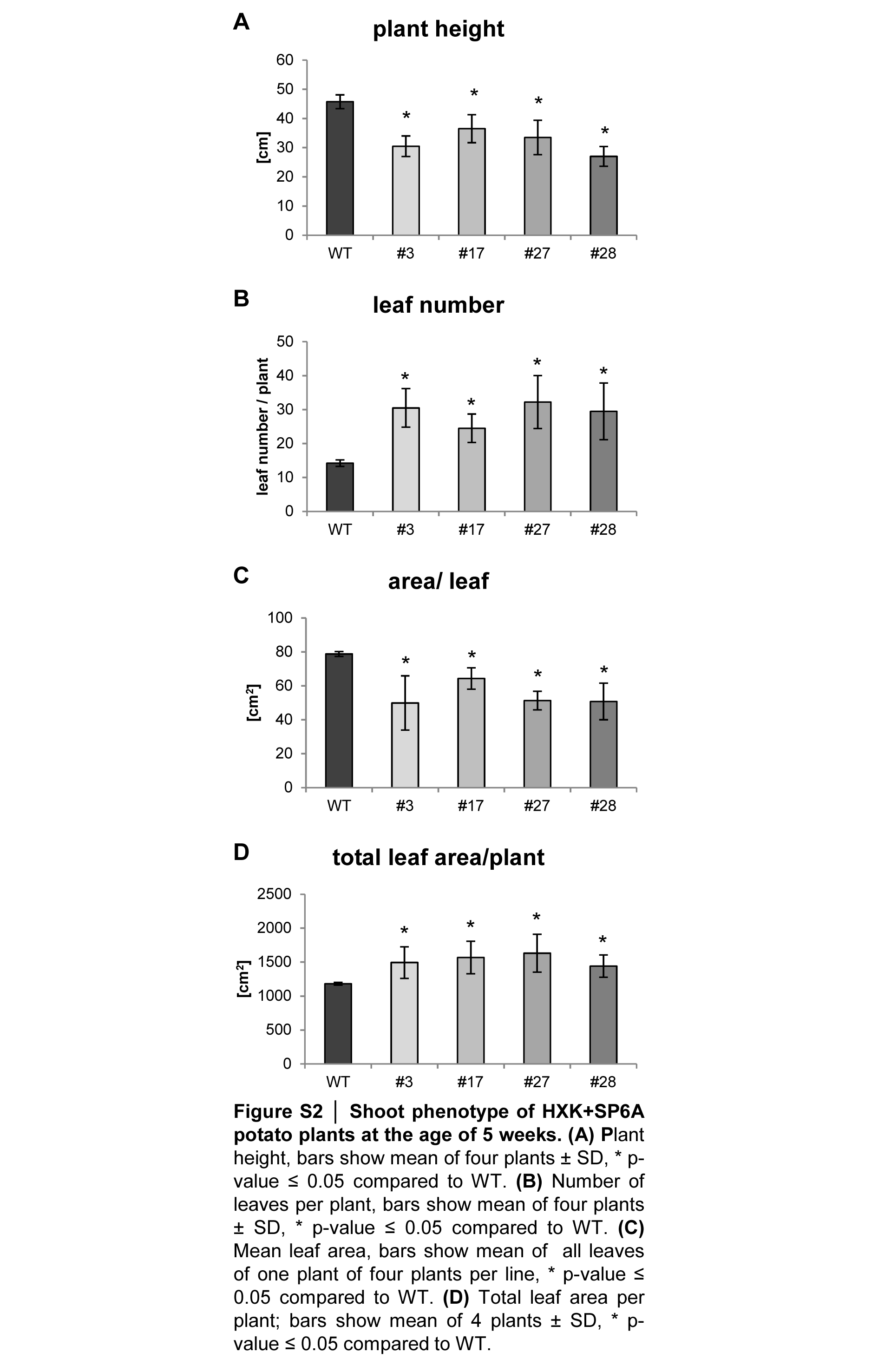

Supplement: Supplementary file 4 [file Image_2.png]

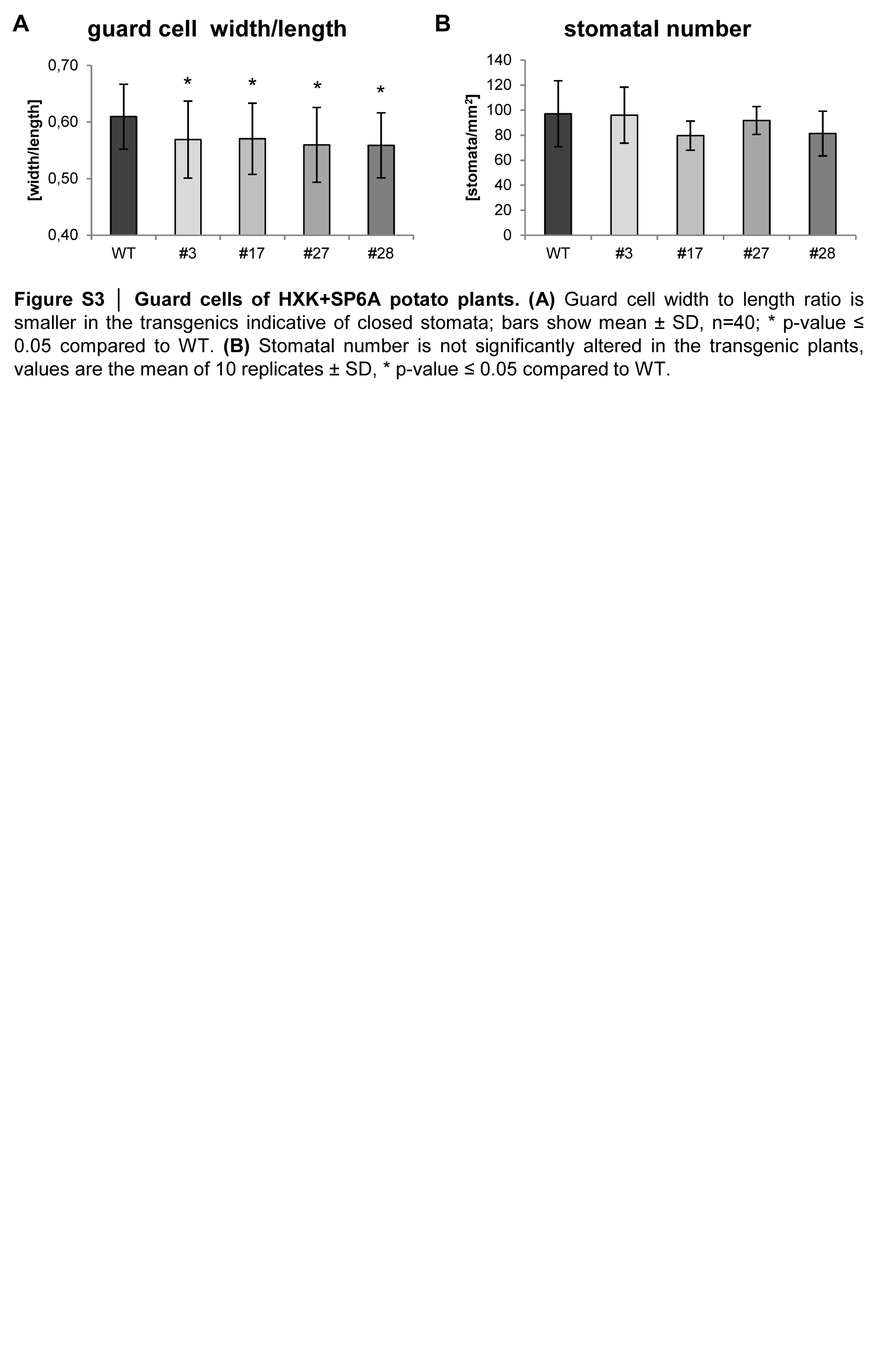

Supplement: Supplementary file 5 [file Image_3.png]

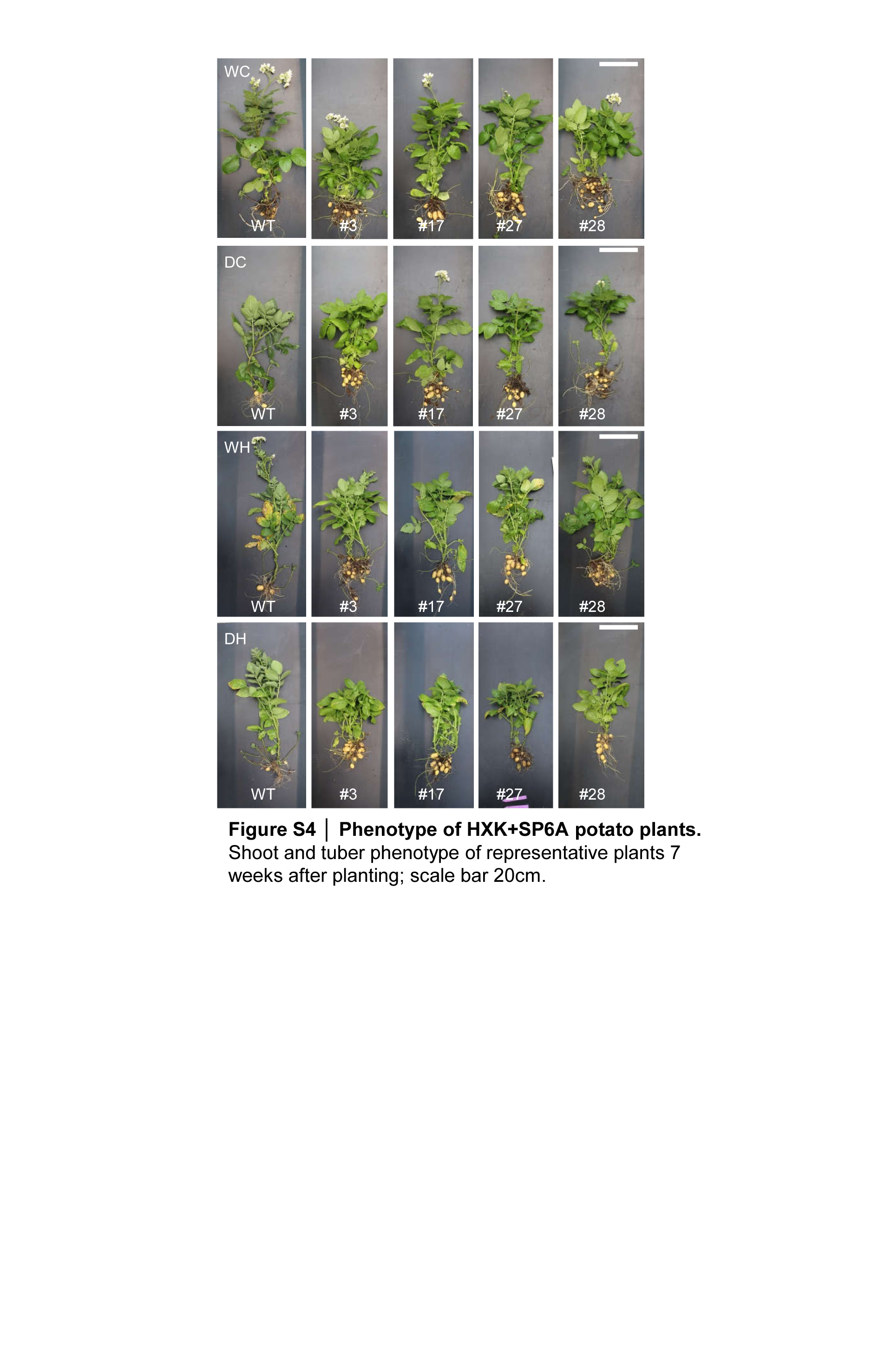

Supplement: Supplementary file 6 [file Image_4.png]

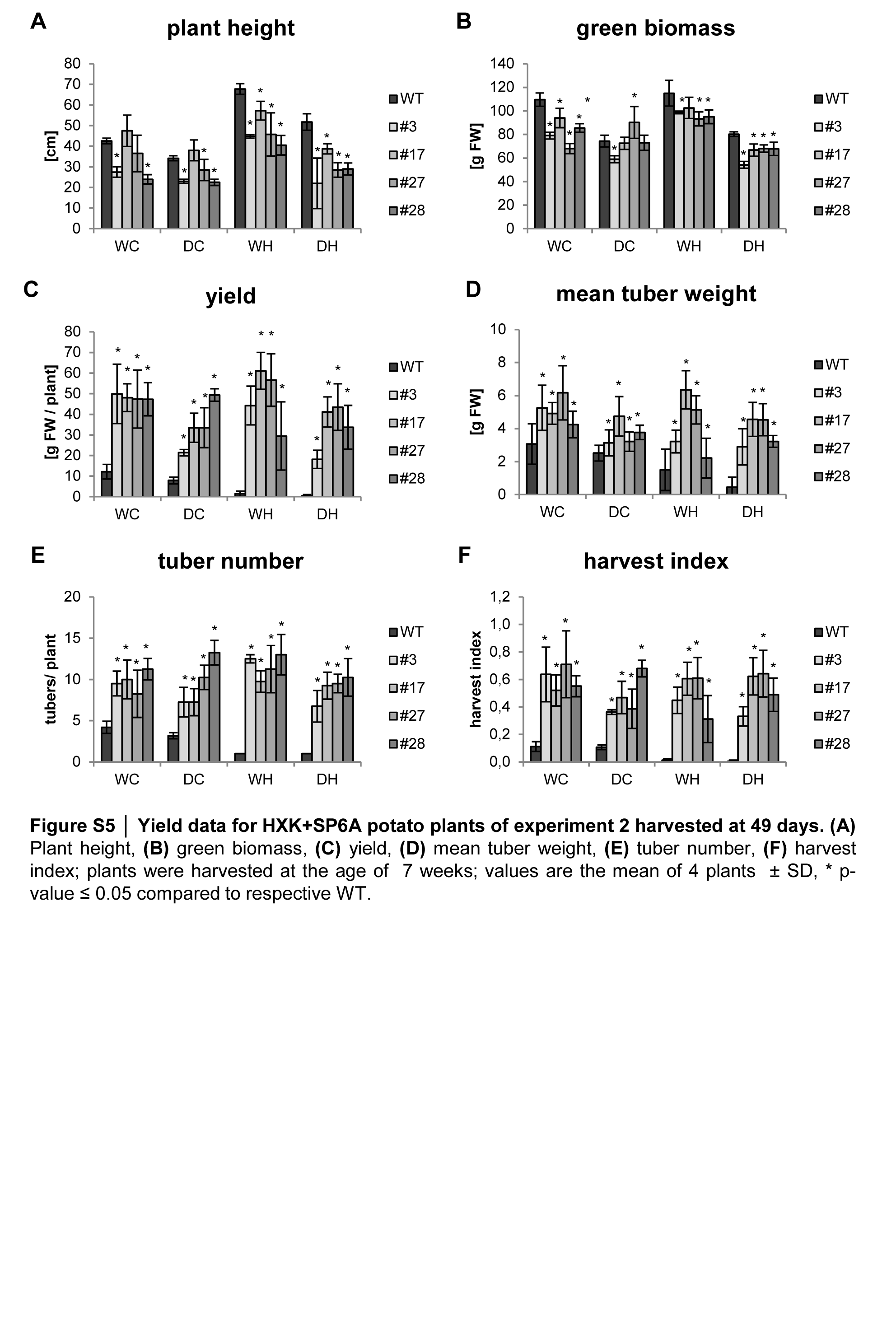

Supplement: Supplementary file 7 [file Image_5.png]

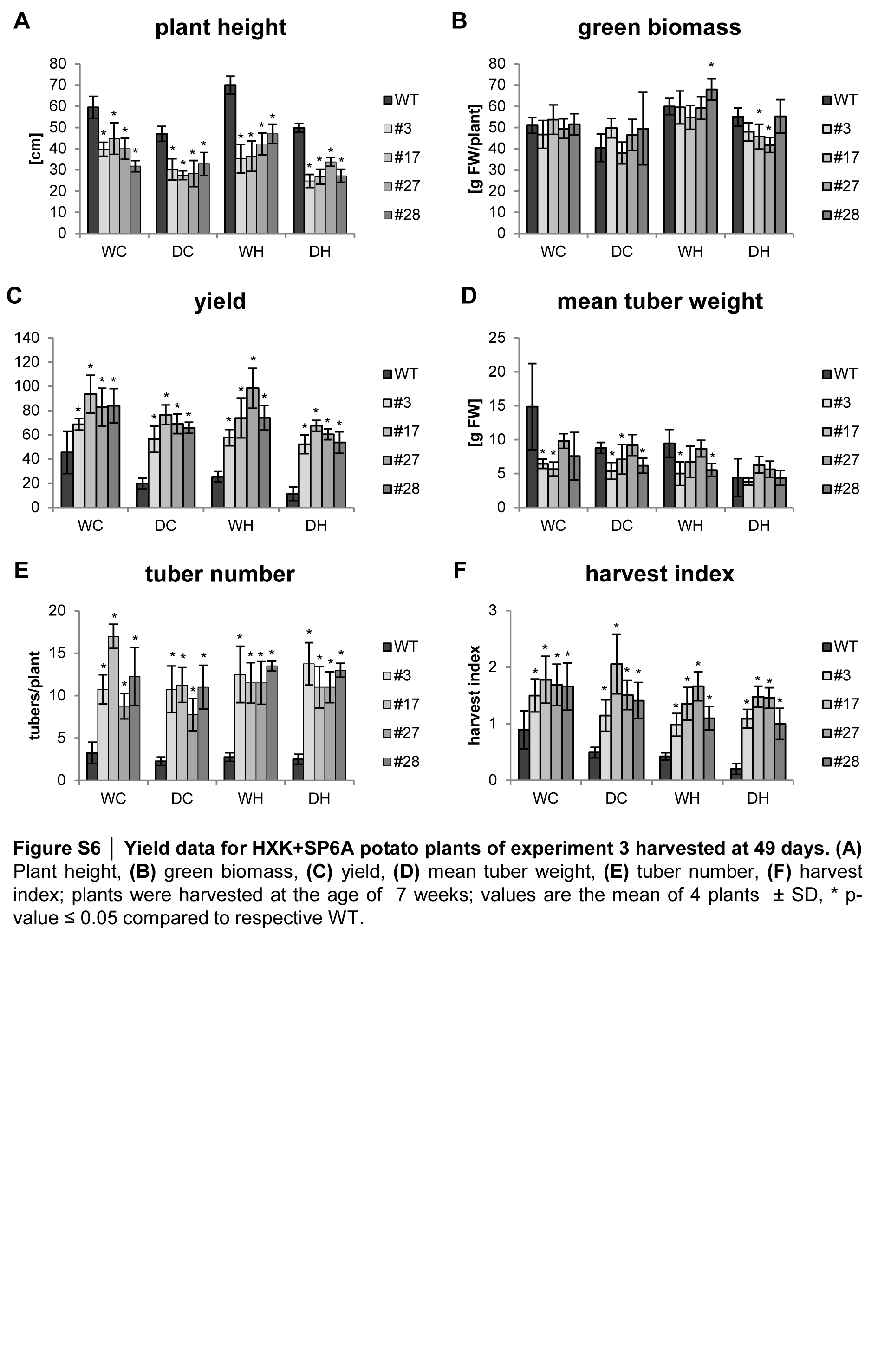

Supplement: Supplementary file 8 [file Image_6.png]

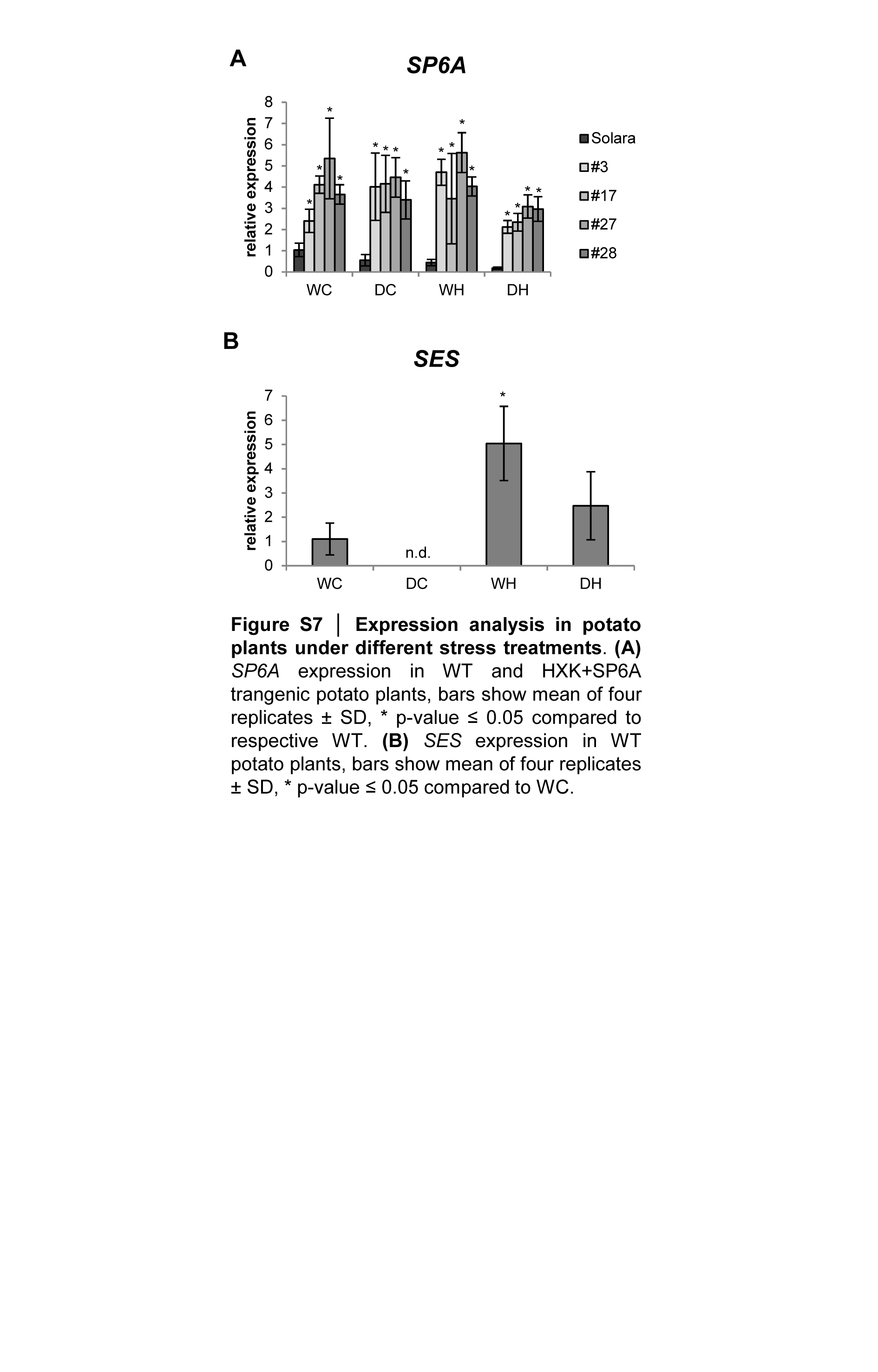

Supplement: Supplementary file 9 [file Image_7.png]
